# Editorial: Antimicrobial resistance genomics in bacterial zoonotic pathogens

**DOI:** 10.3389/fgene.2023.1277081

**Published:** 2023-08-22

**Authors:** Mariela E. Srednik, Mustapha Goni Abatcha, María Laura Chiapparrone

**Affiliations:** ^1^ Veterinary Microbiology and Preventive Medicine, College of Veterinary Medicine, Iowa State University, Ames, IA, United States; ^2^ Veterinary Service Department, Ministry of Agriculture and Natural Resources, Damaturu, Yobe, Nigeria; ^3^ Centro de Investigación Veterinaria de Tandil (CIVETAN), Consejo Nacional de Investigaciones Cientificas y Técnicas, Comisión de Investigaciones Científicas, Facultad de Ciencias Veterinarias, Universidad Nacional del Centro de la Provincia de Buenos Aires, Tandil, Argentina

**Keywords:** editorial, antimicrobial resistance, genomics, bacterial pathogens, zoonotic pathogens

This editorial features a Research Topic of articles published in Frontiers in Genetics and Frontiers in Microbiology: *Antimicrobial resistance genomics in bacterial zoonotic pathogens*. The goal of this Research Topic is to observe the genetic diversity, evolution, and emergence of new clones in a specific population through the analysis of whole-genome sequencing (WGS). The findings of this Research Topic will contribute to the knowledge of zoonotic bacterial pathogens of relevant importance to public health. Antibiotic resistance is a growing global concern due to the emergence of pathogens with multi-drug resistance (MDR) including clinically important antibiotics. MDR strains are a major risk to public health because of the potential for treatment failures particularly for zoonotic pathogens (pathogens that can be transmitted from animals to humans). WGS is a comprehensive method for analyzing entire genomes. Analysis of WGS can give genomic information to characterize strains, detect the presence or absence of resistance genes, plasmids, integrons, and to observe the phylogenetic relationship among a population.

The following are articles published in the *Antimicrobial resistance genomics in bacterial zoonotic pathogens* Research Topic.

*Characterization of cephalosporin and fluoroquinolone resistant Enterobacterales from Irish farm waste by whole genome sequencing* by Prendergast et al. Animal waste can be an important source of antimicrobial resistant bacteria resulting in the spread to soil and water environments (Li et al., 2015). This original study reports the characterization of ESBL, AmpC and *qnr* genes in Enterobacterales collected from untreated and treated farm wastewater in Ireland using whole genome sequencing. Antimicrobial resistant Enterobacteriales can persist in the environment even following wastewater treatment. Enterobacteriales resistant to third generation cephalosporins and fluoroquinolones persist in the environment and carry plasmids that disseminate resistance genes of clinical relevance to the environment. Subsequent propagation could lead to the contamination of drinking water or irrigation water used in crops and ultimately pose a risk to human or animal health. A study of this nature can help support risk assessments on the impact that the environment has on the contamination of foods or on public health that were identified by the European Food Safety Authority (EFSA BIOHAZ Panel et al., 2021).

*Whole-genome sequencing and phylogenetic analysis capture the emergence of a multi-drug resistant Salmonella enterica serovar Infantis clone from diagnostic animal samples in the United States* by Srednik et al. It is important to know that *Salmonella* Infantis is a pathogen that causes foodborne outbreaks, and multidrug resistant isolates are of particular concern due to their resistance to a variety of antimicrobials with limited treatment alternatives. This original report demonstrated the emergence and spread of a *S.* Infantis clone harboring a megaplasmid containing a specific chromosomal mutation conferring resistance to fluoroquinolones, often containing a *bla*_CTX-M-65_ gene coding for an extended-spectrum β-lactamase, and multiple antimicrobial resistant genes. The analysis of *n* = 200 whole genome sequences from veterinary *S.* Infantis isolates submitted at the National Veterinary Diagnostic Laboratories between 2014–2017 captured the emergence of a clonal lineage in 2016 in samples from animals (poultry, cattle, and horses) in the U.S. This clonal lineage harbored a conjugative megaplasmid (pESI-like) with similar antimicrobial resistance pattern, and genetically alike to the ESI clone that emerged in Europe (Franco et al., 2015). Antimicrobial resistance surveillance using whole-genome sequencing in foodborne bacteria population is a useful tool to identify new multidrug resistant bacteria and isolates linked to multidrug resistant clones spreading worldwide (Gymoese et al., 2019).

*Oxazolidinone resistance genes in florfenicol-resistant enterococci from beef cattle and veal calves at slaughter* by Nüesch-Inderbinen et al. Linezolid is an oxazolidinone antimicrobial drug commonly used to treat severe infections in humans caused by multidrug resistant Gram-positive bacteria in intensive care units (Hashemian et al., 2018). Therefore, the emergence of linezolid-resistant bacteria is a threat to public health. In food-producing animals, the use of florfenicol has selected florfenicol-resistant bacteria with specific resistance genes (*cfr*, *optr*A, and *poxt*A) that may confer resistance to oxazolidinone antimicrobials. This research article highlights the occurrence of florfenicol resistant enterococci and other bacterial species carrying linezolid resistance genes (by the use of whole genome sequencing) in beef cattle and veal calves in Switzerland. The presence of clinically relevant resistant Gram-positive bacteria, such as the *optr*A- and *poxt*A-carrying *Enterococcus faecium* ST18, associated with nosocomial infections, emphasizes the potential risk to human health through dissemination of strains carrying oxazolidinone resistance genes into the food chain.

*Genetic characterization of MDR genomic elements carrying two aac(6′)-aph(2″) genes in feline-derived clinical Enterococcus faecalis isolate* by Li et al. The gastrointestinal tracts of animal and humans is colonized by enterococci. Multidrug-resistant *Enterococcus faecalis* frequently cause intestinal infections (Fiore et al., 2019). In this publication, a multidrug-resistant *Enterococcus faecalis* strain and its genetic environment were investigated. *Enterococcus faecalis* strain ESC1 was isolated from the feces of a cat and its complete genome was analyzed by whole genome sequencing and bioinformatics tools. A novel 5.4 kb complex transposon and a resistance plasmid with multiple homologous recombination in *Enterococcus faecalis* ESC1 isolate were reported in this study. This multidrug-resistant strain exhibited a new compound transposon carrying two aminoglycoside resistance genes *aac*(6′)-*aph*(2″). This work provides an understanding of the genomic signature and antibiotic resistance mechanisms of *Enterococcus faecalis* ESC1. It is possible that companion animals could pose a threat to the health of human beings as they can carry diseases and antimicrobial resistant bacteria.
